# Communication Apprehension and Psychological Well-Being of Students in Online Learning

**DOI:** 10.3390/bs11110145

**Published:** 2021-10-23

**Authors:** Somya Agrawal, Shwetha M. Krishna

**Affiliations:** 1 Department of Information Management, Chaoyang University of Technology, Taichung 413310, Taiwan; asomya@gm.cyut.edu.tw; 2 Business School, Vellore Institute of Technology, Chennai 600127, India

**Keywords:** psychological well-being, communication apprehension, perceived learning, social media usage, psychological stress, online learning

## Abstract

The current pandemic has modified how education, learning, and technology interact with one another inside universities. The usage of technology for instructional purposes raises the question of whether learning that happens in an online environment is as effective as traditional classroom models. Within this context, this study explores the psychological well-being of students during the COVID-19 pandemic, using an online cross-sectional survey. Data were collected from 246 university students currently studying at a private university in India. Hierarchical regression analysis and structural equation modelling were used to study the mediating effects between communication apprehension, perceived learning, and psychological well-being under the moderating effects of intention to use social media and psychological stress. Results show that higher intentions to use social media alleviated the negative effects of communication apprehension on perceived learning. Interestingly, it was also found that perceived learning had a significant positive relationship with psychological well-being when students experienced higher levels of psychological stress (eustress). Based on the technology acceptance model (TAM) and the transactional theory of stress and coping, we attempt to integrate the findings related to these theories, which can be considered distinct to previous studies. Implications, limitations, and future directions for research and practice have also been discussed.

## 1. Introduction

Psychological well-being is considered important for university students in order to achieve their goals and fulfill their utmost potential [1]. The past decade has seen an exponential rise in the existence of mental health conditions among university students [2]. Recent research also shows that there is a rising need for help from university students who struggle with significant mental health concerns [3,4,5]. Therefore, examining factors that impact the psychological well-being of university students can help researchers identify ways to train students for a better life [6]. The current pandemic situation has also initiated a global discourse on how education, learning, and technology need to be reimagined in a dynamic world of spiraling complexity, uncertainty, and precarity [7]. Recent research shows that there is an increase in financial and psychological stress in university students, compared to the pre-COVID-19 period [3].

Considering the numerous factors that impact learning and the vast multiplicity of subjects and instructional models, it would be extremely insightful to researchers, practitioners, and educationists to weigh up learning effectiveness and how that impacts the psychological well-being of students across a broad spectrum of instructional methods, experiences, and content areas. This information has become of more interest recently, as the changes taking place in the educational approach differ from the norm, particularly when new instructional methods, such as cutting-edge technologies or computer-mediated communication [8], are being used for the purposes of communication. The usage of technology, particularly social media for instructional purposes, raises the question of whether learning that happens in an online environment is as effective as traditional classroom models. Under these conditions, the role of teachers and students as well as social media for online learning is ambiguous and should be reconsidered [9]. Interestingly, recent research shows that there has been an increase in the usage of social media as a tool for professional communication and education [10]. However, the cons of such platforms have also been considerably discussed in recent studies, presenting mixed results [8]. This skepticism requires more empirical research.

Past research shows that a link exists between communication apprehension and student outcomes [11], in which communication apprehension has been identified as a major drawback to student success, especially in terms of learning [12]. This is associated with more stress and anxiety at the prospect of interacting with fellow class members and teachers. Student stress and anxiety have often been cited as having a negative impact on students’ academic performance [13]. The notion whether social media can play any role in mitigating these challenges still remains unclear. Interestingly, considerable attention has been paid to the influence of learning abilities on academic performance [14], but less attention is afforded on their influence on non-academic outcomes, such as psychological well-being. University students are associated with a higher risk of suffering from mental health conditions, negatively impacting their learning ability and university life experience [15]. Therefore, it is necessary to identify the linkages between communication apprehension, perceived learning, the role of social media, psychological stress, and students’ psychological well-being.

The current study attempts to contribute in this direction in the sense that, firstly, it examines the above-mentioned interrelationships within the context of online education. Secondly, based on the technology acceptance model (TAM) [16] and the transactional theory of stress and coping [17], we attempt to integrate the findings related to these theories in one study, which can be considered distinctive from previous studies. Thirdly, the current study can be considered to be one of the first to associate psychological well-being with perceived learning and communication apprehension, which lacks empirical evidence in the existing literature within the context of online education. Lastly, this pandemic is a call to action for several stakeholders, including researchers, educationists, course instructors, students, and parents, to consider how social media can be incorporated appropriately to maximize its benefits, while recognizing its associated limitations. Therefore, within the context of virtual education, this study aims to examine the following research statements:

1. The interrelationship between communication apprehension and perceived learning of students.
2. The interrelationship between perceived learning and the psychological well-being of students.
3. To examine whether perceived learning mediates the relationship between communication apprehension and the psychological well-being of students.
4. Based on the TAM, to examine whether students’ intention to use social media moderates the interrelationship between communication apprehension and perceived learning.
5. Based on the transactional theory of stress and coping, to examine whether psychological stress moderates the interrelationship between perceived learning and the psychological well-being of students.

To summarize, the remainder of the paper is structured as follows. Section 2 presents the contextual background and literature, in which we present the findings from existing works. Section 3 describes the methodology, data collection procedure, and elaborates on the variables and measurements. In Section 4, we report the results and data analysis. Section 5 presents the discussion of the findings, the implications of the current study, the limitations and future research directions. Section 6 ends with conclusions.

## 2. Background and Literature Review

**Figure 1:**
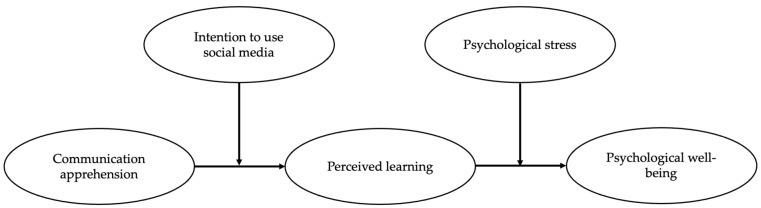
Hypothesized Research Model.

In this section, we set the context of the study and, thereafter, presents the literature of all the variables considered in this paper. The hypothesized research model is depicted in Figure 1.

### 2.1. Setting the Context—Virtual Learning during the COVID-19 Pandemic

There are two distinct approaches to online education: distance learning and online or virtual learning [18]. Online or virtual instruction requires a course instructor to provide instruction synchronously (real-time) or asynchronously (self-paced learning), which offers the students an online forum for open discussion and doubt clarification [19]. This mode of fully online instruction makes use of video conferencing technology and enables the course instructor to deliver classes as they would in a conventional classroom setting. Distance learning also has the component of online study, with access to online learning materials. However, the key distinction is that distance learning also often includes face-to-face workshops, summer classes, or ‘residentials’ as part of the coursework. Therefore, it is more appropriate to call this mode of instruction as blended learning or flexible learning [20].

Dodd et al. [3] found that the COVID-19 pandemic had a huge impact on the studies of students, impeding their overall learning experience. The most common issues were that students found it hard to communicate with other students and teachers online, making it more difficult to learn online as compared to learning face-to-face. Johnson [21] observed that 35% of students reported increased anxiety linked with the transition from face-to-face to virtual learning in the spring 2020 semester, which maps to the early phases of the COVID-19 outbreak. Stress was majorly associated with adapting to online learning methods, which presented particular challenges for individuals who lacked adequate internet access in their homes [22]. This shows that the norms of physical distance present students with fewer opportunities to attend university campuses to maintain social connections, resulting in social fragmentation and isolation, thereby hampering psychological well-being among university students [23].

However, teachers and students in another study reported several advantages to online learning as they found it was less time consuming than face-to-face learning and more comfortable in attending classes in the home environment. They described the benefits of online learning, such as self-paced learning, cost saving, convenience, and flexibility [24]. In 2004, Bernard et al. [25] conducted a meta-analysis of 232 studies, which concluded that there is no average difference in student performance between online and traditional modes of instruction. Similarly, Russell [26], in their compilation of results of over 350 research reports, summaries, and papers, found that virtual education is equally effective as traditional modes of instruction. However, due to the mixed results, the existing evidence is not sufficient and more empirical studies need to be carried out to measure learning effectiveness in technologically mediated instructional practices [27]. Considering the rise in mental health conditions in university students [2], supporting the psychological well-being and learning experience of university students should be of the highest priority during the current pandemic situation as well as in the post-pandemic context. Within this context, the discourse on how education, learning, and social media can be incorporated in virtual classrooms needs to be reimagined to turn technology into a valuable asset for university students while also retaining their psychological well-being [7].

### 2.2. Communication Apprehension and Perceived Learning

Perceived learning can be characterized into three types: cognitive learning [28], affective learning [29], and psychomotor learning [30]. Cognitive learning is associated with recalling knowledge and developing intellectual skills [28]. It is often linked with the ability of paraphrasing, breaking down a problem into smaller units, and problem solving [30]. Affective learning is related to emotions towards the content or subject-matter [29]. Its main focus is on the development of attitudes and behaviors, rather than on the intellectual abilities upon which the cognitive type of learning is based. The third type of learning, i.e., psychomotor learning, is linked with developing physical skills related to manual tasks and movement, operating equipment, such as a computer, and performing in the fields of science, art, and music [30]. Previous research has showcased a clear link between communication apprehension and different types of learning [11]. Communication apprehension is termed as “an individual’s level of fear or anxiety associated with either real or anticipated communication with another person or persons” [31]. It is applicable across many situations and has several recognizable characteristics and reactions [32,33]. Recently, several scholars have become interested in examining communication apprehension within the context of virtual education [34,35,36].

McCroskey and Beatty [37] categorized communication apprehension into trait communication apprehension, generalized-context communication apprehension, individual-group communication apprehension, and situational communication apprehension. Trait communication apprehension is identified as a personality trait of an individual, which is slightly distinct to generalized-context communication apprehension, as the latter is more relevant in certain contexts or situations. Similarly, individual-group communication apprehension is a response reaction to the individual/group with whom the individual is communicating, whereas situational communication apprehension is a response reaction to the situation in which an individual is communicating [38]. In the current study, communication apprehension within the context of classroom was explored. Vu et al. [39] stated that communication apprehension in the classroom is characterized by a certain level of anxiety or fear linked with participating in the classroom. It has a negative correlation with academic achievement [40], cognitive learning [41], affective learning [11], psychomotor skills [42], and communication competence [43], and acts as a main hindrance to student success across various contexts, especially in terms of cognitive, affective, and psychomotor learning outcomes.

It was found that majority of the students refrained from asking questions or making comments during class, while some students feared making presentations [42]. On the other hand, there were some students who dealt with the feeling and eventually brought themselves to communicate despite being fearful, while others preferred waiting for the end of the class to ask questions to the instructors. It was also found that students tried to be discreet, skipped class at times to avoid the feeling of fear, experienced anxiety, and dropped necessary courses [39]. More apprehensive students performed worse academically compared to the students who reported low or moderate levels of communication apprehension [44]. Bourhis and Allen [41], in their meta-analysis, also found that the relationship between communication apprehension and cognitive performance was negative, whereas Messmann and Jones-Corley [11] found that students who reported reduced apprehension levels showed an improvement in affective learning. Given that communication is important in a classroom environment, it is extremely crucial to examine the impact of communication apprehension in order to improve the learning experience of students [45]. This leads us to Hypothesis 1.

**Hypothesis 1** **(H1).** *Communication apprehension is negatively related to perceived learning in students.*

### 2.3. Perceived Learning and Psychological Well-Being

Psychological well-being “is a form of well-being based on the idea of universal human needs and effective functioning” [46]. It demonstrates the awareness of real nature and the realization of the individuals’ human potential. University students have often been associated with high risks of suffering from mental health conditions induced by the academic, social, and financial areas of their lives [47,48]. Mental health conditions, such as stress and anxiety, have shown direct negative impacts on students’ learning abilities and their university life experience [48,49,50]. Due to the increasing number of students suffering from mental health conditions in the past decade [2], it has become extremely important for universities to understand particular stressors to better support students’ psychological well-being [15]. Studies show that when students reflect upon their own learning (referred to as metacognition), it can help to reduce anxiety in classrooms [51] and improve their overall mental well-being (i.e., psychological well-being). In the study carried out by [52], they defined mental well-being as a “positive and sustainable mental state that allows individuals, groups and nations to thrive and flourish”. Oftentimes, it is also defined in terms of happiness, subjective well-being, and eudaimonic well-being in individuals [53].

Diener et al. [46] associated psychological well-being with an individual’s cognitive and affective assessments of his or her life as a whole. Thus, within the university context, an individual’s psychological well-being would include both cognitive perceptions related to university life (i.e., cognitive learning) and emotional reactions to university events (i.e., affective learning). Past studies have made use of different student learning outcomes, such as course grades and GPAs, to evaluate learning effectiveness [54]. However, “their reliability and validity have been questioned because of factors such as grade inflation, which is the tendency to provide higher grades for the same substantive performance at different levels of study or at different periods in time” [55]. The extent to which GPAs could represent students’ actual learning ability may also be skewed. To overcome this limitation, learning outcomes have often been linked to non-academic outcomes, such as psychological well-being, in terms of information reliability and validity, which makes their results even more comparable [46]. Examining the interrelationship between perceived learning and psychological well-being may provide additional insights for the discussion of improving student outcomes in terms of effective learning [48]. This leads us to Hypothesis 2.

**Hypothesis 2** **(H2).** *Perceived learning is significantly related to psychological well-being in students.*

### 2.4. Mediating Effects of Perceived Learning on Communication Apprehension and Psychological Well-Being

Learning is often driven by personal motivations, such as reduced expectations for learning at the start of a course, which can result in a “self-fulfilling prophecy” when the aspect of communication apprehension is added onto the situation [45]. Past research has given sufficient evidence suggesting that apprehensive students are dissatisfied with their experiences in various communication situations [56]. Communication apprehension was often seen as a strong inhibitor of a student’s participation in communication activities in technology mediated classes, not only in traditional classrooms [57,58]. For instance, the existence of inherent communication apprehensive tendencies would affect the nature and frequency of interactions initiated by students in a virtual class. It was found that, when students are characterized by having high levels of communication apprehension, it restrains their learning abilities, digresses interactions with other classmates, and causes social withdrawal [59], thus directly impeding the learning process [60]. It leads to them being more stressed and anxious in classroom discussions, which often becomes accentuated by the fear of negative evaluation and being judged by other students. Therefore, fostering an atmosphere in the classroom in which students are comfortable about sharing their opinions and being wrong can decrease student anxiety and stress [61], communication apprehension [62], and promote psychological well-being.

Downing et al. [63] found that, when students overcome their fear to communicate with other students in class, it provides them with a chance to also alleviate their fear of negative evaluation. Thus, communicating with other students before sharing their ideas with the entire class (called “warm calling”) is beneficial because it frames errors as part of the learning process. This can further reduce stress and contribute to enhanced metacognition, learning for the student [63,64], and improve their overall psychological well-being [65]. Learning happens in a collaborative environment, where an individuals’ apprehension levels act as an important influence on the extent to which they derive intrinsic rewards from the interaction [66]. For example, when students’ communication apprehension levels are above their class average, they would be more likely to report positive intrinsic rewards (i.e., higher levels of perceived learning). However, when the students’ communication apprehension levels are below their class average, the students will experience a decreased level of perceived learning [59]. This is also linked to their psychological well-being in the sense that students with high levels of communication apprehension would experience higher levels of stress, anxiety, be less active in class and participate less, and feel dissatisfied with their learning outcomes [67], which in turn affects their psychological well-being. This leads us to Hypothesis 3.

**Hypothesis 3** **(H3).** *Perceived learning mediates the relationship between communication apprehension and psychological well-being in students.*

### 2.5. Moderating Effects of the Intention to Use Social Media on Communication Apprehension and Perceived Learning

Social media is defined as “interactive computer-mediated technologies that facilitate the creation or sharing of information, ideas, career interests and other forms of expression via virtual communities and networks”. It includes various popular technology platforms, such as Twitter, Facebook, Instagram, LinkedIn, blogging platforms, WeChat, and WhatsApp [8]. The instant messaging applications of Facebook and WhatsApp are the most commonly used social media platforms for social communication and sharing information [68]. In terms of usage, Facebook and WhatsApp do not explicitly compete with each other, and researchers have argued that individuals adopt a wide range of tools on a daily basis [69]. Even though they offer very similar features, the two tools involve different social practices, leading to different user experiences with the same functionalities [70]. For example, Facebook provides better support for multitasking in asynchronous communication practices, while in WhatsApp’s confined environment users experience a deep sense of presence in the act of communication. Social media platforms accessed using mobile or web-based technologies create highly collaborative platforms in the form of content sharing sites, blogs, social networking, and wikis [71]. Past research underlines that social media and its features offer individuals a unique experience capable of overcoming challenges faced during face-to-face interaction [32]. Moreover, individuals consider that they derive more benefits from using social media to communicate, which makes the medium seem like the optimal method of communication compared to other methods [72].

Davis [16] proposed a widely accepted theory that is concerned with examining usage behavior of a new technology, called as the technology acceptance model (TAM). Due to its simplicity (parsimony), data supportability (verifiability), and applicability to predict acceptance and usage of new technologies in various contexts (generalizability), the TAM has been very popular for decades [73,74,75]. According to the TAM, the intention to use social media determines an individual’s behavior, showcasing whether the person will use the technology or not. Intention to use social media is the discretionary and cognitive depiction of the user’s readiness to actually use social media. Rauniar et al. [75] propose that the intention to use social media is determined by the user’s perceived benefit from social media. They concluded that an individual engages in a social-media-related activity, reaps the benefits, and develops a future intention to use the activity. This further leads to more engagement with the social media site, which is consistent with the intentions formed from past usage. This causality helps to explain the heavy usage of social media sites, such as Facebook and WhatsApp [10,76].

Therefore, individuals facing difficulties in communication due to their communication apprehension tend to prefer computer-mediated communication (CMC) over face-to-face communication [77]. Social media offers individuals more control over interaction and their non-verbal cues and therefore provides a better opportunity to engage in a more careful self-presentation [78]. It offers interactive and effective communication to discuss easily, exchange information, and share knowledge. It also reduces the psychological distance between students and teachers [79]. In terms of affective learning, social media provides students a psychological safety environment where they usually engage in open discussion, speak up, and listen actively [80]. When students can express themselves freely without fear of judgment, their learning will improve [81,82]. Reduced communication apprehension increases students’ learning. Based on this information, it seems that communication through a technology-mediated channel would offer students, especially those with higher levels of communication, more opportunities to participate more in class discussions in order to meet their needs for interaction [32]. Moreover, the automated and dynamic feedback that teachers and peers give students in online learning environments can provide a more personalized learning experience to students, thereby increasing their motivation, engagement, and learning [83]. This leads us to Hypothesis 4.

**Hypothesis 4** **(H4).** *The intention to use social media moderates the relationship between communication apprehension and perceived learning such that a negative relationship is weaker for students with a higher intention to use social media as compared to students with a lower intention to use social media for communication.*

### 2.6. Moderating Effects of Psychological Stress on Perceived Learning and Students’ Psychological Well-Being

Learning is a non-linear form of logical problem solving [84], which happens in a non-complex environment. Therefore, considering contextual, social, and emotional factors is extremely important to comprehend and explain student behavior in different learning situations. Past studies have shown that learning in the classroom has been strongly influenced by the cognitive theory of stress proposed by Lazarus and Folkman (1984). According to Lazarus and Folkman [85], “psychological stress is a particular relationship between the person and the environment that is appraised by the person as taxing or exceeding his or her resources and endangering his or her well-being”. Moreover, according to their transactional theory of stress and coping [85,86], they propose that stress is a by-product of a transaction between an individual (which includes cognitive, physiological, affective, psychological, and neurological systems) and the complex environment. Therefore, when there is a disparity between perceived task demands and perceived resources to meet these demands (measured in terms of perceived learning), students associate negative emotions to the experience and have a change of mood, which overall affects their well-being [17,85]. The most developed part of the brain is called as the prefrontal cortex, which is associated with higher-order thinking and decision making. Research shows that this region of the brain is the most affected by stress [83]. Such disablement to the prefrontal cortex could “display as difficulties with impulse control, impaired memory retrieval, and difficulties with executive skills such as planning, problem-solving, and monitoring errors” [83]. This showcases that psychological stress affects learning due to an interplay between cognitions and emotions.

Frijda [87] connected emotions as direct responses to the individual’s current concerns. The concerns are the perceptions of the individuals that turn a situation into a satisfier (linked with positive cognitions and emotions) or into an annoyer (problematic situation that may cause harm). Therefore, when an individual experiences strong emotion, such as stress, against a particular situation in class, it raises concerns for their mental well-being. It requires an extra processing capacity for toning down emotions and for tuning back in on the task [84]. Bower [88] also demonstrated that emotions may have a strong impact on the information processing ability of individuals. For example, when a student is in a sad mood or feeling stressed, he or she spends more time reading and re-reading negative details about the topic. This makes it harder for the student to improve his or her learning. On the contrary, a student who is in a positive mood will spend more time encoding positive details and later recall more positive things about the topic. This will in turn further his or her learning. Therefore, it is extremely crucial for students to be aware of their emotions and moods. This leads us to Hypothesis 5.

**Hypothesis 5** **(H5).** *Psychological stress moderates the relationship between perceived learning and psychological well-being such that the interrelationship is weaker for students with a higher level of psychological stress as compared to students with a lower level of psychological stress.*

## 3. Methodology and Data Collection

### 3.1. Data Collection Procedure

The data for the current study were collected at a private university in India. The convenience sampling method was used to select the sample. The students had enrolled for undergraduate and postgraduate programs in different departments at the university. A total of 580 self-administered survey questionnaires were distributed online to students who had registered for courses, such as entrepreneurship and development, ethics and values, and human resource management. The participation of the respondents was made voluntary and the section related to demographic information was prepared anonymously. The respondents were ensured that the collected data would be kept confidential and solely used for research purposes. The questionnaires were distributed to different classes in order to increase the response rate and ensure that the probability that students participating in the survey did not know each other was high. We received a total of 246 usable responses after keeping the online questionnaire survey link open for one week, which resulted in a response rate of 42.4%. The final sample consisted of 158 male and 88 female students. Out of all the students, 172 students were from undergraduate courses and 74 students were from postgraduate classes. The age of the students ranged between 17–23 years, and the average age was 19.5 years. A total of 136 students were from the engineering major (55%), 39 students from the law major (16%), and 71 students from the management major (29%). A total of 75 students were from dual-earner families and 171 were from single-earner households.

### 3.2. Variables and Measures

The measures used for each construct have been explained in detail below, along with the Cronbach’s alpha value. All the items for variables, such as perceived learning, communication apprehension, psychological well-being, and intention to use social media, were rated using the Likert-type 5-point scale, with ‘1’ indicating ‘strongly agree’ and ‘5’ indicating ‘strongly disagree’ in the questionnaire items. The items for the variable of psychological stress were rated using a different Likert-type 5-point scale, with ‘1’ indicating ‘always’ and ‘5’ indicating ‘never’ in the questionnaire items. Previous studies have confirmed good psychometric properties for all the constructs measured in the current study.

#### 3.2.1. Perceived Learning

This construct was measured using four items adapted from the CAP Perceived Learning Scale [89]. This scale is reliable to measure the three dimensions of perceived learning, such as perceived cognitive, affective, and psychomotor learning in online courses. A five-point Likert scale was used, with which the students were required to rate statements such as, “I can organize content learned from the online courses into a logical structure” and “I feel more self-confident as the result of the content learned in the online courses”. The coefficient alpha (α) value for the scale was estimated to be 0.811.

#### 3.2.2. Communication Apprehension

The items for this construct were adapted from the PRCA-24 instrument scale [90]. This instrument was chosen based on the different contexts to which it can be applied [38]. This instrument is considered highly relevant in examining communication behaviors in individuals. In the current study, four items were used for the measurement, with which the students were required to rate statements such as, “I feel tensed and nervous while engaging in group discussions online with new people” and “I feel afraid to express myself or ask doubts during online classes”. The value for alpha (α) reliability of this scale was estimated to be 0.749.

#### 3.2.3. Psychological Well-Being

The construct of psychological well-being was inspired from the Warwick–Edinburgh mental well-being scale to monitor and improve mental well-being in individuals [91]. In their validation study, it focused on the positive aspects of mental health and did not showcase any ceiling effects in a varied population sample. We used four items to measure this construct, with which the students were required to rate statements, such as “I’ve been feeling optimistic about the future” and “I’ve been feeling useful after attending the course”. The coefficient alpha (α) value for the scale was 0.787.

#### 3.2.4. Intention to Use Social Media

The technology acceptance model (TAM) explains that “individuals’ performance of a specified behavior is determined by their behavioral intention to perform a certain task” [16]. Based on this theory, three items for the intention to use technology were adapted from the TAM, which have received a large amount of empirical support for being robust and parsimonious in predicting technology acceptance and adoption within individuals. The students were required to rate statements, such as “I intend to use Facebook/WhatsApp for communicating with my classmates” and “I intend to use Facebook/WhatsApp to be reconnected with people that matter to me”. The value for alpha (α) reliability of this scale was estimated to be 0.833.

#### 3.2.5. Psychological Stress

The perceived stress scale (PSS) is considered to be a classic stress assessment instrument to measure how different situations affect an individual’s feelings and their perceived stress [92]. Four items were used to measure this construct, with which the students were asked questions about their feelings and thoughts during the last month. For example, the students were required to rate statements, such as “In the last month, how often have you felt that you were unable to control the important things in your life?”. In each case, the students were asked to indicate how often they felt or thought a certain way. The coefficient α value for the scale was 0.648.

#### 3.2.6. Control Variables

We included the demographic variables of age and gender in the questionnaire. The variable of gender of the respondent was coded as “1” = male and “2” = Female. The students were also asked whether their parents were working. The respondents were required to respond as ‘Yes/No’, and the variable was coded as “1” = Yes and “2” = No.

## 4. Results and Data Analysis

This section presents the results and data analysis.

### 4.1. Descriptive Statistics

Table 1 presents the descriptive statistics and Pearson correlation values for the measured constructs. The constructs of communication apprehension show negative correlations with intention to use social media, perceived learning, and psychological well-being. Similarly, the intention to use social media is positively correlated with perceived learning, psychological stress, and psychological well-being.

### 4.2. Measurement Model

Table 2 summarizes all the model fit indexes. As shown in the table, the model fit indexes of the measurement model justify further examination of the structural model (χ^2^/DF = 1.717, *p* ≤ 0.001; CFI 0.94, GFI = 0.91, SRMR = 0.058, RMSEA = 0.054).

### 4.3. Structural Model

**Figure 2:**
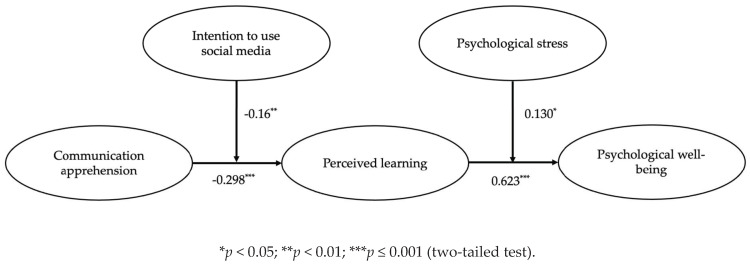
SEM model with the results of the moderation analysis.

Figure 2 shows the overall structural model with path coefficients. The results suggest that the hypothesized model fit the data well (χ^2^/DF = 2.88, *p* ≤ 0.001; CFI = 0.95, GFI = 0.94, SRMR = 0.058, RMSEA = 0.060). Hypothesis 1 states that communication apprehension is negatively related to students’ perceived learning. Our results support this view (β = −0.298, *p* ≤ 0.001). Hypothesis 2, which states perceived learning is positively related to psychological well-being in students, was also supported by the results (β = 0.623, *p* ≤ 0.001). Similarly, Hypothesis 3 states that perceived learning mediates the relationship between communication apprehension and psychological well-being. We used the Sobel test to verify the indirect effects in the hypothesized model [93]. The results confirm the indirect negative effects of communication apprehension on psychological well-being through perceived learning (z = −3.530, *p* ≤ 0.001). To test the mediating effects, we also inspected three alternative models that are plausible on the basis of theoretical arguments. As shown in Table 2, these models provide an adequate fit to the data, but are not better than the hypothesized model, demonstrating the mediating effects of perceived learning.

### 4.4. Hierarchical Multiple Regression Analysis

**Figure 3:**
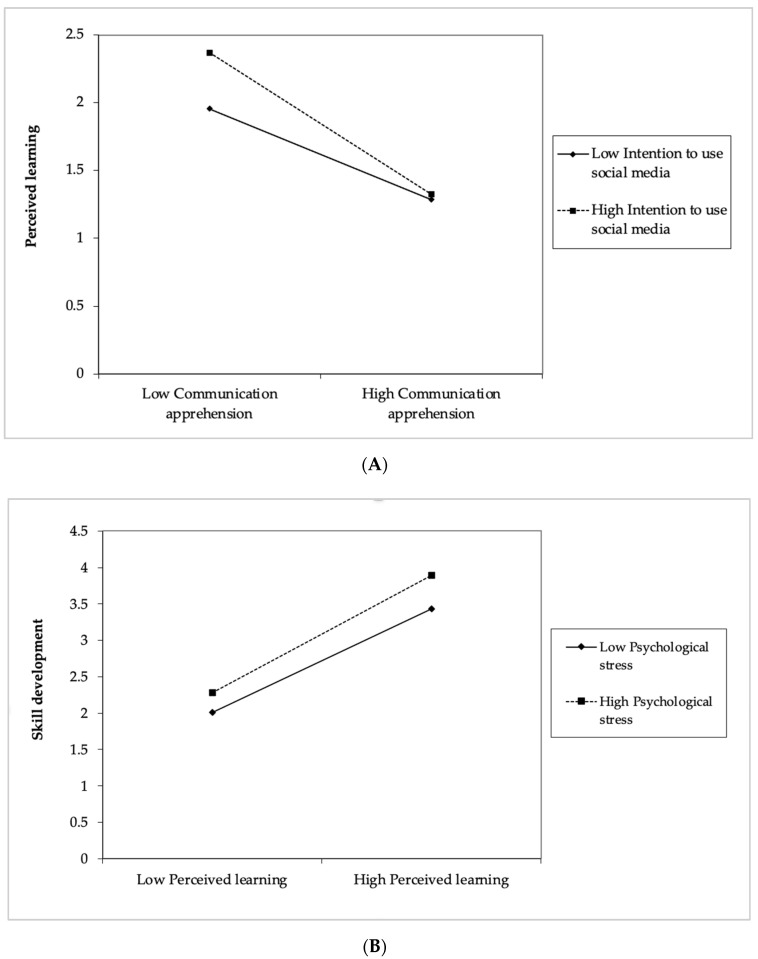
Moderating Effects: (**A**)—Moderating effects of the intention to use social media on communication apprehension and perceived learning; (**B**)—Moderating effects of psychological stress on perceived learning and psychological well-being.

Table 3a summarizes the hierarchical multiple regression results. In keeping with Hypothesis 4, we found that the intention to use social media significantly and negatively moderated the relationship between communication apprehension and perceived learning (β = −0.16, *p* ≤ 0.05). However, the plot presented in Figure 3A suggests that, although a higher level of communication apprehension is associated with a lower perceived learning, students’ higher intentions to use social media in class alleviated the negative effects of communication apprehension on their perceived learning.

Referring to Table 3b, the argument of Hypothesis 5 is that stress interacts significantly with perceived learning to positively influence psychological well-being. The results support this argument (β = 0.130, *p* ≤ 0.05). The plot (Figure 3B) suggests that higher levels of perceived learning are associated with higher levels of psychological well-being in the presence of lower as well as higher levels of psychological stress. Selye, in the study ‘The Stress Concept: Past, Present and Future’ [94], proposed that stress response can be differentiated into both negative and positive aspects, known as distress and eustress. This justifies the plot, showcasing that some amount of stress is good for the students and facilitates positive outcomes. Interestingly, traditional conceptions about stress determines it as inherently dysfunctional; however, psychological theory suggests that it is not intrinsically maladaptive [95].

## 5. Discussion

This paper reports the psychological well-being of university students in India at a time when there were substantial changes in universities due to the onset of the COVID-19 pandemic. The shift to online learning had a significant impact on the learning experience of students and their psychological well-being. The current study examined the links between communication apprehension, perceived learning, and learning outcomes when moderated with the intention to social media and psychological stress. Consistent with previous findings, the results of the current study show that communication apprehension is negatively related to students’ perceived learning [39]. University students’ fear that their thoughts or views will not be accepted by their classmates when they participate in the classroom. Therefore, the students tend to participate within the acceptable limits to avoid breaking classroom norms and being rejected by their peers [96]. To mitigate the negative effects, teachers could examine whether technology usage can motivate students’ towards participating in class and building positive self-concepts [97] to promote psychological well-being within virtual class room settings.

Secondly, perceived learning was found to be positively related to a student’s psychological well-being. Past research supports this result [46], as psychological well-being is associated with an individual’s cognitive perceptions related to university life (i.e., cognitive learning) as well as emotional reactions to university events (i.e., affective learning). Mahmoudzadeh et al. [98] also found that learning and developing cognitive and affective skills are intertwined with the psychological well-being of students. In another study, Pietarinen et al. [99] suggest that efficiency in dealing with the demands of study are interrelated with the psychological well-being experienced by students. Thus, course instructors could make use of various interventions based on regulating cognitive and affective strategies, which can be useful in monitoring psychological well-being among students.

Thirdly, we found that perceived learning reduced the negative effects of communication apprehension on psychological well-being through mediation. This is in line with previous findings. Students who showcase higher levels of affective and cognitive learning are more efficient in dealing with their studies and display more persistence when facing problems, such as psychological stress and anxiety, compared to students who suffer from reduced levels of emotional and cognitive learning [100]. During the learning process, when students overcome their fear to communicate with other students [63], they feel comfortable in sharing their opinions, reducing their anxiety and stress levels [61], and enhance their psychological well-being. Therefore, course facilitators could encourage students to participate in communication activities, which may further help students to develop confidence in their learning abilities. This can contribute to enhance their perceived learning outcomes and thereby increase their willingness to participate in future classroom activities [101].

Fourthly, we found that the intention to use social media was able to reduce the negative effects of communication apprehension on perceived learning. This is congruous with the findings of previous studies, which have shown that students with high levels of communication apprehension (i.e., shy individuals) felt less of communication apprehension during the discussion conducted online compared to face-to-face communication [77]. Due to the availability of several social media platforms (such as Facebook and WhatsApp), students now have the option to choose the mode of communication that makes them feel comfortable in their communication or complement their lack of communication skills [10]. Joinson [78] found that individuals disclose several details about themselves, in a more sustained manner, online and are also more willing to reveal less socially desirable information in online communication settings compared to face-to-face contexts. This might be due to the heightened anonymity and the reduced non-verbal and/or demographic cues that social media provides compared to face-to-face communication [102]. However, our results show that the negative effects of communication apprehension on perceived learning still exist to some extent even during online communication, which shows that online communication makes shy people experience lesser communication apprehension; however, it does not make them feel completely confident about their communication abilities [8,77].

Lastly, the results show that psychological stress moderated the relationship between perceived learning and psychological well-being. This is consistent with past research because psychological stress has been continuously associated with poor learning performance [49,103,104]. Mental health issues have often been negatively linked with students’ educational experience, leading to a low GPA score and reduced graduation and retention rates [50]. In turn, poor academic performance has been found to affect the psychological well-being of students [105,106,107]. Moreover, Grubic et al. [108] pointed out that the online learning might also cause more psychological stress for university students who have already experienced heightened levels of mental health problems and undermined psychological well-being during the COVID-19 pandemic [109].

### 5.1. Implications of the Current Study

Considering the wide variety of factors that might influence learning in the presence of different instructional models, it would be helpful to both researchers and practitioners to compare learning effectiveness across a broad spectrum of instructional experiences and content areas [110]. Such a cross-categorical approach to measure learning effectiveness would prove helpful when different instructional modes, such as online, blended, and face-to-face instruction, but also to compare different educational tools, techniques, and models to gauge which instructional design works better for the varied content and student populations [111]. However, the challenge lies in measuring learning independently of the course structure, teacher, department, grade level, and other restricting factors. Such information can encourage teachers to make use of online technology tools, such as social media, in designing online courses [89]. The classroom implications of the results of the current study involve both course instructors and students.

Firstly, the course instructors might benefit from incorporating new methods of instruction that involves making use of social media [70]. Doing so would be advantageous to students suffering with and above average communication apprehension for their successful collaborative learning with other classmates [59]. Taking insights from the findings of the current study, the course instructors could also consider designing various options for task completion and learning modalities that involve communication through social media [15]. Providing such alternatives would allow students to manage their communication expectations and apprehension levels, without affecting their stress levels [112]. Secondly, there is a common misapprehension with respect to online learning that assumes that students are not provided additional support or academic help to complete the courses. This is not completely true, as course instructors and online tutors provide support to students synchronously as well asynchronously undertaking the courses. Due to the convenience of instant messaging features in social media platforms [69], the instructors and tutors can be contacted via Facebook or WhatsApp when required [18].

### 5.2. Limitations and Future Research

The current study is not without limitations. Firstly, the data were collected only once, incorporating a cross-sectional research design. This limits the scope of the findings as the gradual development in variables was not observed. Future studies could benefit from examining the temporal aspects of the interrelationships tested in the current study. Secondly, we made use of a self-report instrument to measure perceived learning in students. Due to this, there might be a possibility of potential conflation of factors in the student’s view of the educational experience, in terms of cognitive and affective learning. Even though the nature of psychomotor learning (related to skills) is a comparatively straightforward deliberation, it might be arduous for students to separate their cognitive learning from their affective conceptions, especially when they are in the process of completing a course. Future research can implement the CAP Perceived Learning Scale across a broad variety of disciplines and contexts (for example, employees seeking online training for various professional reasons), which can provide more empirical evidence to further validate the measurement scale or create the opportunity for reiterative improvement. Thirdly, the data of the current study were collected from undergraduate students, so the findings cannot be generalized to other contexts (for example, organizations). Future studies can broaden the scope to other contexts and collect data accordingly (for example, collect data from organizations). Fourthly, with respect to social media usage, issues, such as maintaining privacy of information, transparent communication, and building trustworthiness among students while information sharing, were not covered in the current study. Moreover, personal views, inherent prejudices, and erroneous information can be challenging to diagnose when we are overwhelmed by a large volume of evolving social media content. Therefore, future studies can benefit from including such aspects in their research and also highlight the importance of monitoring the information exchanged for quality and reliability, and respect confidentiality of information shared when participating in online education. Lastly, the current study was carried out in India; therefore, the results need to generalized for other geographies with caution. Future studies can consider replicating the objectives of the study in other regions to examine distinctions in student behavior in different geographical regions.

## 6. Conclusions

Due to the growing concerns related to the psychological well-being of university students’ during the online education period, it has become imperative for researchers, practitioners, and educationists to take measures to help students survive the non-traditional learning experiences and maintain psychological well-being. Within the context of online education, the results of the current study show the mediating effects of perceived learning and how they positively impact psychological well-being in students, especially those who are bound by communication apprehension. We also studied the moderating effects of the intention to use social media and the psychological stress on the interrelationships, and found interesting results. Through this study, we contend that it is not possible to fully conclude that the pandemic due to COVID-19 was the main reason to impact variances in psychological well-being, or whether the results found in the current study related to psychological well-being along with other interrelationships could have been detected before the pandemic. However, the findings in the current study identify key factors of communication apprehension, the role of social media, and psychological stress across students associated with their perceived learning and psychological well-being during the pandemic. They highlight the importance for universities, policymakers, and course instructors to make use of social media and adapt the current instructional practices to meet the educational and emotional goals of university students and provide support to these groups now to avoid aggravating existing disparities.

## Figures and Tables

**Table 1:** Means, standard deviations, and correlations.

SNo.	Variables	Mean	S.D.	1	2	3	4
1	Communication apprehension	2.74	0.832				
2	Intention to use social media	1.916	0.701	−0.079			
3	Perceived learning	3.1	0.811	−0.228 **	0.090		
4	Psychological stress	2.81	0.458	0.309 **	0.147 *	−0.091	
5	Psychological well-being	2.97	0.796	−0.249 **	0.009	0.554 **	−0.053

Notes: *n* = 246 * *p* < 0.05; ** *p* < 0.01 (two-tailed test).

**Table 2:** Summary of model fit indexes.

Model Test	χ^2^	df	SRMR	CFI	GFI	RMSEA
**Independence model**	1674.53	125				
**Measurement model**	214.59	125	0.058	0.941	0.913	0.054
**Hypothesized model**	96.08	51	0.058	0.954	0.941	0.060
**Alternative model 1 ^a^**	101.52	52	0.061	0.949	0.938	0.062
**Alternative model 2 ^b^**	166.402	52	0.137	0.883	0.905	0.095

Note: ^a^: Remove direct link between communication apprehension and psychological well-being; ^b^: Swap orders between perceived learning and psychological well-being.

**Table 3:** Results of the hierarchical regression analysis.

**(a) Moderating effects of the intention to use social media on communication apprehension and perceived learning ^a,b^**			
**Variables**	**Model 1**	**Model 2**	**Model 3**
Control variables			
Age	−0.055 (0.028)	−0.050 (0.028)	−0.047 (0.027)
Gender	−0.085 (0.109)	−0.061 (0.106)	−0.046 (0.107)
Parents working	−0.067 (0.114)	−0.053 (0.111)	−0.073 (0.106)
Communication apprehension		−0.213 *** (0.07)	−0.210 *** (0.07)
Intention to use social media		0.079 (0.075)	0.060 (0.07)
Communication apprehension X Intention to use social media			−0.160 ** (0.07)
ΔR^2^		0.054	0.024
F for R^2^		6.813 ***	6.355 **
R^2^	0.015	0.049	0.070
F	1.24	3.50 **	4.046 ***
**(b) Moderating effects of psychological stress on perceived learning and psychological well-being ^a,c^**			
**Variables**	**Model 1**	**Model 2**	**Model 3**
Control variables			
Age	−0.059 (0.03)	−0.028 (0.02)	−0.030 (0.02)
Gender	−0.110 (0.10)	−0.063 (0.09)	−0.080 (0.09)
Parents working	−0.053 (0.11)	−0.017 (0.09)	0.006 (0.09)
Perceived learning		0.543 *** (0.05)	0.571 *** (0.05)
Psychological stress		−0.004 (0.09)	−0.000 (0.09)
Perceived learning X Psychological stress			0.130 * (0.04)
ΔR^2^		0.291	0.015
F for R^2^		49.86 ***	5.39 *
R^2^	0.019	0.309	0.325
F	1.514	21.228 ***	18.91 ***

^a^*n* = 246. Values are standardized coefficients, with standard errors in parentheses. ^b^ Perceived learning is the dependent variable. ^c^ Psychological well-being is the dependent variable. * *p* ≤ 0.05 ** *p* ≤ 0.01 *** *p* ≤ 0.001.

## Data Availability

The data presented in this review are available on request from the corresponding author.
